# Thiosulfate sulfurtransferase deficiency promotes oxidative distress and aberrant NRF2 function in the brain

**DOI:** 10.1016/j.redox.2023.102965

**Published:** 2023-11-19

**Authors:** Yang Luo, Laurent Chatre, Shaden Melhem, Zayana M. Al-Dahmani, Natalie Z.M. Homer, Anneke Miedema, Leo E. Deelman, Matthew R. Groves, Martin Feelisch, Nicholas M. Morton, Amalia Dolga, Harry van Goor

**Affiliations:** a University of Groningen, Department of Molecular Pharmacology, Groningen Research Institute of Pharmacy, Faculty of Science and Engineering, Groningen, the Netherlands; b University Medical Center Groningen, Department of Pathology and Medical Biology, Groningen, the Netherlands; c Université de Caen Normandie, CNRS, Normandie University, ISTCT UMR6030, GIP Cyceron, F-14000 Caen, France; d Centre for Cardiovascular Science, Queen's Medical Research Institute, University of Edinburgh, Edinburgh, United Kingdom; e University of Groningen, Department of Pharmacy, Drug Design, Groningen, the Netherlands; f Mass Spectrometry Core, Edinburgh Clinical Research Facility, University of Edinburgh/BHF Centre for Cardiovascular Sciences, Queen's Medical Research Institute, University of Edinburgh, Edinburghh, United Kingdom; g University of Groningen, University Medical Center Groningen, Department of Clinical Pharmacy and Pharmacology, Groningen, the Netherlands; h Clinical and Experimental Sciences, Faculty of Medicine, University of Southampton and University Hospital Southampton NHS Foundation Trust, Southampton, United Kingdom; i Centre for Systems Health and Integrated Metabolic Research, School of Science and Technology, Nottingham Trent University, Nottingham, United Kingdom

**Keywords:** Thiosulfate sulfurtransferase (TST), Reactive species, Antioxidant, Oxidative distress, Nrf2 signaling

## Abstract

Thiosulfate sulfurtransferase (TST, EC 2.8.1.1) was discovered as an enzyme that detoxifies cyanide by conversion to thiocyanate (rhodanide) using thiosulfate as substrate; this rhodanese activity was subsequently identified to be almost exclusively located in mitochondria. More recently, the emphasis regarding its function has shifted to hydrogen sulfide metabolism, antioxidant defense, and mitochondrial function in the context of protective biological processes against oxidative distress. While TST has been described to play an important role in liver and colon, its function in the brain remains obscure. In the present study, we therefore sought to address its potential involvement in maintaining cerebral redox balance in a murine model of global TST deficiency (*Tst*^−/−^ mice), primarily focusing on characterizing the biochemical phenotype of TST loss in relation to neuronal activity and sensitivity to oxidative stress under basal conditions. Here, we show that TST deficiency is associated with a perturbation of the *reactive species interactome* in the brain cortex secondary to altered ROS and RSS (specifically, polysulfide) generation as well as mitochondrial OXPHOS remodeling. These changes were accompanied by aberrant Nrf2-Keap1 expression and thiol-dependent antioxidant function. Upon challenging mice with the redox-active herbicide paraquat (25 mg/kg i.p. for 24 h), *Tst*^−/−^ mice displayed a lower antioxidant capacity compared to wildtype controls (C57BL/6J mice). These results provide a first glimpse into the molecular and metabolic changes of TST deficiency in the brain and suggest that pathophysiological conditions associated with aberrant TST expression and/or activity renders neurons more susceptible to oxidative stress-related malfunction.

## Graphical abstract

**Figure:**
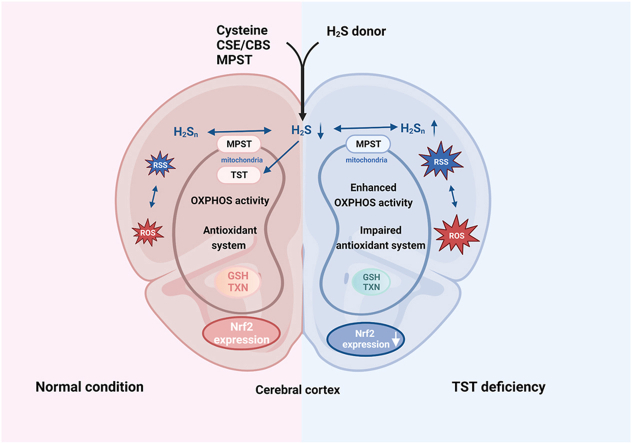

## Abbreviations

TST: Thiosulfate sulfurtransferase
CBS: Cystathionine beta synthase
CSE: Cystathionine gamma lyase
MPST: 3-mercaptopyruvate sulfurtransferase
H_2_S: Hydrogen sulfide
SQOR: Sulfide quinone oxidoreductase
PDO: Persulfide dioxygenase
DHLA: Dihydrolipoic acid
GSH: Glutathione
BMI: Body mass index
HFD: High-fat diet
SOD: Superoxide dismutase
ROS: Reactive oxygen species
2-PTS: 2-propenyl thiosulfate
DSS: Dextran sulfate sodium
RNS: Reactive nitrogen species
RSS: Reactive sulfur species
RSI: Reactive species interactome
GSSG: Glutathione disulfide
GPX: Glutathione peroxidase
GR: Glutathione reductase
FCCP: Carbonyl cyanide-*p*-trifluoromethoxyphenylhydrazone
TMPD: N,N,N′,N'-tetramethyl-*p*-phenylenediamine
PRDX: Peroxiredoxin
NOS: Nitric oxide synthase
CAT: Catalase
NRF2: Nuclear factor erythroid 2-related factor 2
KEAP1: Kelch-like ECH-associated protein 1
HMOX1: Heme Oxygenase 1
TXN: Thioredoxin
NQO1: NAD(P)H dehydrogenase [quinone] 1
GCLC: Glutamate cysteine ligase, catalytic subunit
GCLM: Glutamate cysteine ligase, modifier subunit
GSTM1: Glutathione S-transferase Mu

## 1 Introduction

The enzyme thiosulfate sulfurtransferase (TST, EC 2.8.1.1) was first identified in 1933 as a cyanide (CN^−^) detoxification enzyme that utilizes thiosulfate (S_2_O_3_^2−^) as sulfur donor to form thiocyanate (SCN^−^; also known as rhodanide) [1,2]. The use of thiosulfate as an antidote to cyanide poisoning relies on this enzymatic reaction. Another known rhodanese enzyme, 3-mercaptopyruvate sulfurtransferase (MPST, EC 2.8.1.2), shares a high sequence homology with TST, and both enzymes are thought to be evolutionarily closely related [3,4]. TST is abundantly expressed in various tissues and plays a vital role in three important biological processes: the metabolism of hydrogen sulfide (H_2_S; hereinafter referred to as sulfide), modulation of mitochondrial respiratory chain activity, and involvement in the thiol-dependent antioxidant system [[5], [6], [7], [8]].

In the context of sulfide metabolism, TST oxidizes the endogenous gasotransmitter H_2_S in a similar fashion as sulfide quinone oxidoreductase (SQOR) and persulfide dioxygenase (ETHE1/PDO) [9]. TST also has the capacity to form H_2_S from thiosulfate by utilizing dihydrolipoic acid (DHLA), the reduced form of α-lipoic acid [10,11]. Balancing both functions, H_2_S oxidation and production, it seems that the primary function of TST is in the detoxification of H_2_S and less so in the production of H_2_S [12]. TST is almost exclusively expressed in mitochondria, the organelle that synthesizes ATP by oxidative phosphorylation (OXPHOS) through stepwise reduction of oxygen in the Electron Transport Chain (ETC) and the generated proton gradient using five enzyme complexes (Complex I–V) [13,14]. TST activity appears to modulate reduced nicotinamide adenine dinucleotide (NADH) dehydrogenase (Complex I) and other mitochondrial complexes including succinate dehydrogenase (Complex II) via interaction with their iron-sulfur clusters [6,7]. In addition to this interaction between mitochondrial activity and TST, phosphorylation of TST may modulate its activity and thereby affect the stability of sulfur in the iron-sulfur centers of Complexes III and IV, further impacting electron transport and ATP production [15]. In mammalian cells, TST is linked to two thiol-dependent antioxidant systems, the thioredoxin system and the GSH system [15]. TST exerts anti-oxidative properties via donating sulfur for these two systems and an increase of TST activity can (in)directly activate their reactive oxygen species (ROS) scavenging function [12,16].

Oxidative stress has classically been defined as an imbalance in the generation of pro-oxidant and antioxidant species in favor of the former [17]. A series of experimental observations promoted a redefinition of oxidative stress as a condition that is associated with alterations in redox signaling and control, and an updated interpretation of the original concept distinguishes physiological oxidative stress (or ‘oxidative eustress’) from excessive and deleterious oxidative stress (coined ‘oxidative distress’) [18]. Besides ROS, some other types of short-lived molecules such as reactive nitrogen (RNS) and reactive sulfur species (RSS) contribute to cellular signaling processes and merit consideration specifically in the context of sensing and adaptation to changes in environmental and/or metabolic conditions. These considerations are part of the stress signaling paradigm and have recently been conceptualized in the form of the Reactive Species Interactome (RSI) framework [19,20].

The transcription factor Nrf2 (nuclear factor (erythroid-derived 2)-like 2) is a master regulator of cellular homeostasis that controls the expression of genes related to redox homeostasis [21]. The primary mechanism of NRF2 regulation is at the protein level. The majority of research has focused on the involvement of the electrophile and redox sensor Kelch-like ECH-associated protein 1 (KEAP1) in regulating NRF2 protein levels in response to metabolic demands [22]. The association between KEAP1 and NRF2 is disrupted by electrophilic alteration or oxidation of cysteine thiols in KEAP1, enabling cells/organisms to respond to environmental stress. Following dissociation from KEAP1, NRF2 escapes destruction and targets antioxidant response element (ARE) genes, which leads to an enhancement of antioxidant capacity [23]. Moreover, various studies linked the TST-related antioxidant system to nuclear factor erythroid 2-related factor 2 (Nrf2) signaling due to its transcriptional activation of GSH-related enzymes, one of the first lines of defense against oxidative stress [[24], [25], [26], [27]]. Here, H_2_S mediates direct persulfidation of KEAP1 (Kelch-like ECH-associated protein 1) and thereby contributes to sulfide-mediated NRF2 regulation [28].

Several studies have demonstrated that TST provides an essential protective function against disease [15]. Studies investigating the mechanistic effects of TST, which were identified in polygenic lean mouse adipocytes, have indicated its potential as a predictor for diabetes [26]. In an Icelandic cohort of almost 700 people, an inverse correlation was observed between *TST* mRNA levels in subcutaneous adipose tissue and body mass index (BMI) [29]. Upregulation of adipose TST was detected in lean mice challenged with a high-fat diet (HFD), further supporting its likely protective effects [29]. Additionally, mice with transgenic *Tst* overexpression in mature adipocytes, showed resistance against HFD-induced obesity and exhibited higher protein expression of mitochondrial superoxide dismutase 2 (SOD2) and higher mRNA levels of peroxiredoxin 3 (*Prdx3*) compared to control mice, while cytosolic superoxide dismutase 1 (SOD1) remained unchanged [29]. These findings provide evidence of an interaction between TST and mitochondrial ROS. Consistent with a function of TST for ROS removal, knockdown of *Tst* in the preadipocyte cell line 3T3-L1 generated higher levels of mitochondrial ROS in the presence of oxidative stress stimuli [29]. Furthermore, ROS-related adiponectin release from 3T3-L1 adipocytes was decreased in cells treated with the TST inhibitor, 2-PTS (2-propenyl thiosulfate), while adiponectin was increased following addition of the TST substrate thiosulfate [29]. TST enables H_2_S detoxification in colon tissue, and has reduced expression in colon mucosa of patients with ulcerative colitis and colon cancer [[30], [31], [32]]. During the development of dextran sulfate sodium (DSS) induced colitis in mice, the expression of *Tst* mRNA, protein and activity significantly decrease [33].

Taken together, these findings indicate that TST has an important function in maintaining the redox balance in colon and adipocytes, but there are no studies elucidating the importance of TST in redox signaling within the brain. A clinical report of rhodanese (i.e. TST) deficiency in a rare neurological and mitochondrial human disorder known as Leber's Hereditary Optic Atrophy (LHON) [34] prompted us to investigate the underlying molecular and metabolic changes of TST deficiency in the brain. From physiology to pathology, the brain consumes about 20% of the whole-body supply of oxygen and energy [26]. The cerebral cortex, with its intense neuronal activity and high metabolic activity, has a particularly high susceptibility to perturbation by oxidative stress [[35], [36], [37]]. In Alzheimer's Disease, increased oxidative stress is associated with the progression of the neurodegenerative pathology along with amyloid and hyperphosphorylated tau [38,39]. This is due to high mitochondrial generation of ROS, including superoxide anion (O_2_^•^^−^) and hydrogen peroxide (H_2_O_2_) [19,40]. Hence, we hypothesized that TST plays a vital role in enabling an appropriate redox balance in the brain, and that global gene silencing of TST in mice would provide novel insights into the central role of TST in neurological antioxidant signaling and tissue protection.

## 2 Methods

### 2.1 Experimental animals

All the experiments were performed according to international guidelines set out by the ethical committees of the University of Edinburgh and within the framework of the Animals (Scientific Procedures) Act (1986) of the United Kingdom Home Office. Animals were maintained in standard housing conditions with a 12-h light and 12-h dark cycle (7 a.m.–7 p.m.) and *ad libitum* access to rodent chow. All studies used female mice housed in cages of 3–6 individual littermates. The female mice used for the study originated from C57BL/6 N *Tst*^−/−^ mice [29] backcrossed onto a C57BL/6J genetic background for more than 10 generations. Pieces of brain cortex were snap frozen and stored at −80 °C until use.

### 2.2 Induction of oxidative stress *in vivo* using paraquat

To test the efficacy of paraquat dichloride (PQ, 36541-SIGMA) as an inducer of oxidative stress in the brain cortex tissue *in vivo*, PQ (in physiological saline 0.9%) was administered to WT and *Tst*^−/−^ mice by intraperitoneal injection at a concentration of (25 mg/kg body weight). 24 h later, mice were culled by decapitation and organs were harvested for analysis.

### 2.3 Total ATP, GSH/GSSG and MPST activity measurements

Total ATP content was measured with the CellTiter-Glo 2.0 Luminescent assay (G9241, Promega). GSH/GSSG content and ratio were measured with the Glutathione GSH/GSSG Assay Kit (MAK440, Sigma-Aldrich) following the manufacturer's instructions. MPST enzyme activity was detected with MPST ELISA kit (MBS9912889, Mybiosource) according to manufacturer's instructions. All analyses were performed using freshly extracted total protein from brain cortex samples, and using microplate multimode reader Spark (Tecan). For MPST enzyme activity, absorbance reading were obtained by a Synergy H4 Hybrid Reader (Biotek).

### 2.4 Protein extraction and immunoblotting

Protein extraction from brain cortex samples was performed using 50 mM Tris⋅HCl pH 7.5, 150 mM NaCl, 1% (v/v) Triton X-100, 0.1% (w/v) SDS. Extracted proteins were centrifuged to remove sample debris. The protein content was determined with the Bicinchoninic acid (BCA) Protein Assay (23,227, ThermoFisher Scientific/Pierce). 20 μg of protein was loaded for SDS/PAGE. After blotting, *Trans*-blot Turbo nitrocellulose membranes (Bio-rad) were stained with Ponceau S staining solution for protein loading reference pictures, then cut for different membrane immunostaining, and blocked with blocking buffer (5% (w/v) milk in 1 × Tris-buffered saline (TBS), 0.1% (v/v) Tween) for 1 h. Blocked membranes were incubated with primary antibodies in blocking buffer overnight at 4 °C, and finally with HRP-conjugated secondary antibodies for 1 h at room temperature. Detection was performed using Clarity Western ECL substrate (Bio-Rad) and Odyssey Fc Imager (LI-COR). Representative immunoblots are shown. Primary antibodies include TST (HPA003643, Atlas Antibodies), MPST (Ab224043, Abcam), GPX4 (SAB4300725, Sigma-Aldrich), OXPHOS complexes (total OXPHOS Rodent WB Antibody Cocktail, AB110413, ABCAM), SOD1 (HPA001401, Sigma-Aldrich), SOD2 (HPA001814, Sigma-Aldrich), KEAP1 (MABS5164, Millipore), NRF2 (AB62352, ABCAM).

### 2.5 Mitochondrial isolation and high-resolution respirometry

Approximate 50 mg of brain cortex from C57BL/6J and *Tst*^−/−^ mice were harvested and mitochondria were isolated utilizing a semi-automated pump-controlled tissue rupture system (0.7 mL/min) (PCC) and syringes (SGE, Trajan© Scientific, Australia) attached to a cell homogenizer (Isobiotech, EMBL Heidelberg, Germany) installed on a pump (ProSense, Oosterhout, NL) [41]. The isolated mitochondria were suspended in isolation buffer (250 mM sucrose, 20 mM HEPES, 3 mM EDTA, pH 7.5). The mitochondrial concentration was quantified by Pierce® BCA Protein Assay Kit (Thermo Scientific). All the experiments were conducted on ice.

The mitochondrial respiration parameters were analyzed using an O2K oxygraphy (Oroboros systems, Innsbruck, Austria). 250 μg crude mitochondria from wildtype and *Tst*^−/−^ mouse cortexes were monitored under continuous stirring at 750 rpm in 1 mL MiR05 (0.5 mM EGTA, 3 mM MgCl2, 60 mM lactobionic acid, 20 mM taurine, 10 mM KH_2_PO_4_, 20 mM HEPES, 110 mM d-Sucrose, BSA, 1 g/l essentially fatty acid free, at pH7.4). Oxygen polarography was performed at 37 °C and the oxygen flux per tissue mass (pmol O_2_/s/mg) were recorded in real-time using the DatLab5 software. The mitochondrial respiration was assessed with the use of different substrates as follows: 5 mM pyruvate, 2 mM malate and 1.5 μM FCCP to uncouple all the ETC complexes and measure complex I respiration; subsequently, 0.5 μM rotenone was added to inhibit complex I, followed by 10 mM succinate to measure complex II respiration. In addition, 2.5 μM antimycin A was added to prevent both complex I and II mediated respiration due to inhibition of complex III, followed by 2 mM ascorbate combined with 0.5 mM TMPD to measure complex IV-mediated respiration. The measurement sequence was completed by injection of 10 mM sodium dithionite.

### 2.6 Detection of the ROS O_2_^•-^ and H_2_O_2_, RNS NO and ONOO^−^, and the RSS H_2_S and H_2_S_n_

Fluorescent molecules were used to measure ROS, specifically O_2_^−^ (DHE, D7008, Sigma-Aldrich) and H_2_O_2_ (DCF-DA, D6883, Sigma-Aldrich), the RNS NO (DAF-2DA AB145283, ABCAM) and ONOO^−^ (DHR123, D1054, Sigma-Aldrich), and the RSS H_2_S (7-Azido-4-Methylcoumarin, 802,409, Sigma-Aldrich) and H_2_S_n_ (Sulfane Sulfur Probe 4/SSP4, SB10-10, Tebubio) in freshly extracted total proteins from brain cortex samples. This was done by spectrofluorometry using a microplate multimode reader Spark (Tecan).

### 2.7 Total SOD activity, catalase activity, total nitric oxide synthase activity, and total peroxidase activity

Total SOD activity was measured with the Superoxide Dismutase Activity Assay Kit (AB65354, ABCAM), which assesses the inhibition activity of xanthine oxidase by SOD. Catalase activity was assessed with the Catalase Activity Assay Kit (AB83464, ABCAM). Total Nitric Oxide Synthase activity was measured with the Nitric Oxide Synthase (NOS) Activity Assay Kit (MAK407, Sigma-Aldrich). Total peroxidase activity was assessed with the Peroxidase Activity Assay Kit (MAK092, Sigma-Aldrich). Analyses were performed following manufacturer's instructions, with freshly extracted total proteins from brain cortex samples, and using microplate multimode reader Spark (Tecan).

### 2.8 Glutamate content

Glutamate concentration was measured with the Glutamate Assay Kit (AB138883, ABCAM).

Analysis was performed following manufacturer's instructions, with freshly extracted total proteins from brain cortex samples, and using microplate multimode reader Spark (Tecan).

### 2.9 RNA isolation, cDNA synthesis and quantitative real-time PCR

RNA was extracted from tissue slices according to the instruction of miRNeasy ® Mini Kit (Qiagen, Germany). Total RNA concentration was quantified by Nanodrop® (ND-1000 Spectrophotometer). Equal amount of cDNA synthesis and amplification were performed using Superscript II kit and RANDOM primers (Thermo Fisher Scientific, USA), according to the manufacture's protocol. The cDNA synthesis mix are incubated as follows: 10 min at 25 °C, 50 min at 42 °C, 15 min under 70 °C via a Veriti 96 Well Thermal Cycler (Thermo Fisher Scientific). For qRT-PCR, 500 ng of cDNA samples were mixed with SYBR green, forward primers, and reverse primers (Primers used for quantification are shown in Supplementary Table S1). The qPCR was run on LightCycler® 480 II (Roche) and the protocol was as follows: 2 min at 50 °C, 10 min at 95 °C, 40 cycles of 15 s 95 °C and 60 s 60 °C, and the dissociation curve is run under 15 s 95 °C, 15 s 60 °C, 15 s 95 °C. RT-qPCR data was analyzed on LightCycler 480 software. Expression levels were normalized to GAPDH and displayed as 2-delta Ct.

### 2.10 Thiosulfate measurements in the cortex using LC-MS/MS

Frozen cortex (∼25 mg, exact weight recorded) was homogenised in 160 mM EPPS, 16 mM DTPA and monobromobimane, pH 8.0 buffer (300 μL) and acetonitrile (300 μL) with 46 mM monobromobimane (MBBr) (25 μL) for 30 min, room temperature in the dark. The reaction was stopped with ethyl acetate (1 mL), the mixture centrifuged (400×*g* for 15 min at 4 °C), and the upper organic layer (∼800 μL) passed through a 96-well Filter + Plate (Biotage, Sweden), along with an MBBr-derivatised calibration curve of thiosulfate (0, 0.01, 0.025, 0.05, 0.1, 0.25, 0.5, 1, 2.5, 5, 10, 20, 90 and 100 μM), followed by PLD + extraction (Biotage, Sweden), reduction to dryness and resuspension in water (100 μL). Samples were analyzed on an I-Class UPLC system (Waters, UK) on a 16 min chromatographic run using an HSS T3 (2.1 × 150 mm; 1.8 mm) column kept at 50 °C. MBB-thiosulfate was eluted using water (0.1% formic acid)/methanol (0.1% formic acid) system at 0.4 mL/min from 0 to 100% methanol over 6 min, re-equilibrating to 0% methanol at 15 min. Multiple reaction monitoring parameters for the thiosulfate MBB derivative was: *m*/*z* 303.0 → 205.1 on a QTrap 6500+ (AB Sciex) with the following compound settings: declustering potential −65 V, collision exit potential −15 V, collision energy −18 V. Source conditions were −4.5 kV ion spray voltage, 600 °C temperature, gases (GS1 = 40, GS2 = 60, Curtain gas = 40 units). Analyst ® v1.7 was used for instrument control and data acquisition. Data analysis was carried out using MultiQuant v3.0 (AB Sciex). Linear regression analysis of the peak areas of MBB-thiosulfate was used to calculate the quantity of thiosulfate in each sample, corrected for tissue mass.

### 2.11 Statistics

The data are presented as mean ± standard deviation (SD). Statistical differences between two conditions were calculated using an unpaired T-test. Statistical differences among more than two conditions were calculated using an one-way ANOVA test. Statistical analysis was performed in GraphPad Prism (version 9.1.0), and repeat numbers are specified in the figure legends.

## 3 Results

### 3.1 *Tst*^−/−^ cerebral cortex exhibits a steady H_2_S level and a dysregulated GSH pathway

**Fig. 1:**
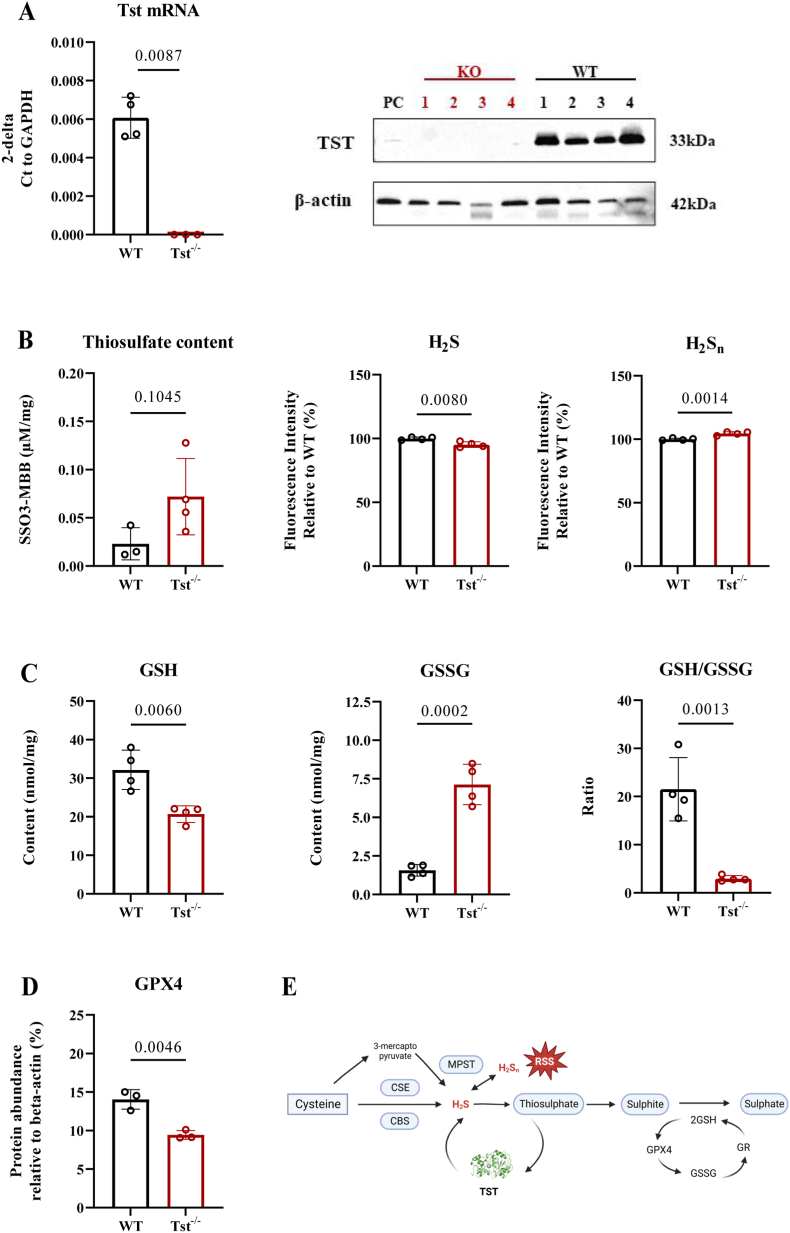
*Tst*^−/−^ mice brain cortexes display a dysregulated GSH pathway. A–D: Data are expressed as mean ± SD, student T test. (A) *Tst* mRNA and Tst protein levels of *Tst*^−/−^ (red) and C57BL/6J (black) mice (n = 4). (B) Thiosulfate content in C57BL/6J mice (Black, n = 3) and *Tst*^−/−^ mice brain cortexes (Red, n = 4) measured by HPLC-MS/MS. H_2_S and H_2_S_n_ concentrations were measured using fluorescent probes, and show that H_2_S decreased 5% and H_2_S_n_ increased 5% in the *Tst*^−/−^(red) compared with C57BL/6J mice (black) (n = 4). (C) GSH and GSSG contents in mice cortexes in the presence (black) or absence (red) of TST (n = 4). (D) Quantification of immunoblots of cerebral cortical area for GPX4 protein level in *Tst*^−/−^(red) compared with C57BL/6J mice (black) (n = 3). The original blotting picture is shown in Fig. S1F. (E) Brief schematic showing H_2_S enzymatic pathway. Sulfide is an endogenously produced gaseous signaling molecule via CBS, CSE and MPST. Polysulfides (H_2_S_n_) became novel reactive sulfur species, which is derived from H_2_S with MPST. TST converts thiosulfate produced via non-enzymatic reactions to sulphite. Then oxidation of sulphite to sulfate is dependent on the glutathione system. GPX4 and GR is the cofactor of conversion between GSH and GSSG. (For interpretation of the references to colour in this figure legend, the reader is referred to the Web version of this article.)

To investigate how TST influences the GSH pathway in the brain, we performed studies in the cortical brain region of conventional knock-out mice in which *Tst* deletion (termed *Tst*^−/−^) has been successfully validated (Fig. 1A). Although the difference in thiosulfate concentrations in the cerebral cortex between wildtype and knockout mice did not reach statistical significance (likely due to small sample size), mean thiosulfate concentration was approx. 3-fold higher in *Tst*^−/−^ mice compared to WT (Fig. 1B). Next, sulfide and polysulfide concentrations were assessed in the cortex. While tissue steady-state levels of both, H_2_S and H_2_S_n_ were found to be maintained at similar levels in *Tst*^−/−^ and C57BL/6J mice, mean concentrations were lower for H_2_S and higher for polysulfides (Fig. 1B). To investigate whether MPST can compensate for the lack of TST in the brain of the *Tst*^−/−^ mice, we analyzed *Mpst* mRNA and protein levels. MPST protein and *Mpst* mRNA was decreased in *Tst*^−/−^ mice (Fig. S1A–D). Interestingly, the MPST activity was not changed, although it tended to be higher in the brain of *Tst*^−/−^ mice compared to C57BL/6J mice (Fig. S1E). To verify whether the GSH pathway was altered in the brain of *Tst*^−/−^ mice, we measured its GSH and GSSG content. GSH was 36% lower in *Tst*^−/−^ mice, while its oxidized form, GSSG, increased 5 times, causing the GSH/GSSG ratio to decline 7.2-fold in *Tst*^−/−^ mice compared to wildtype controls (Fig. 1C). This indicates a redox imbalance secondary to TST enzyme activity loss. Moreover, we found a significant decrease of GPX4 protein (Fig. 1D; Fig. S1F) and the transcriptional level of GR significantly decreased in *Tst*^−/−^ brain cortex (Fig. 4J). Altogether, our results established that expression and activity of TST exert a major effect on both H_2_S metabolism and the GSH antioxidant pathway (Fig. 1E).

### 3.2 TST deletion increases ATP in the cerebral cortex and elevates ROS via modulation of OXPHOS activity

**Fig. 2:**
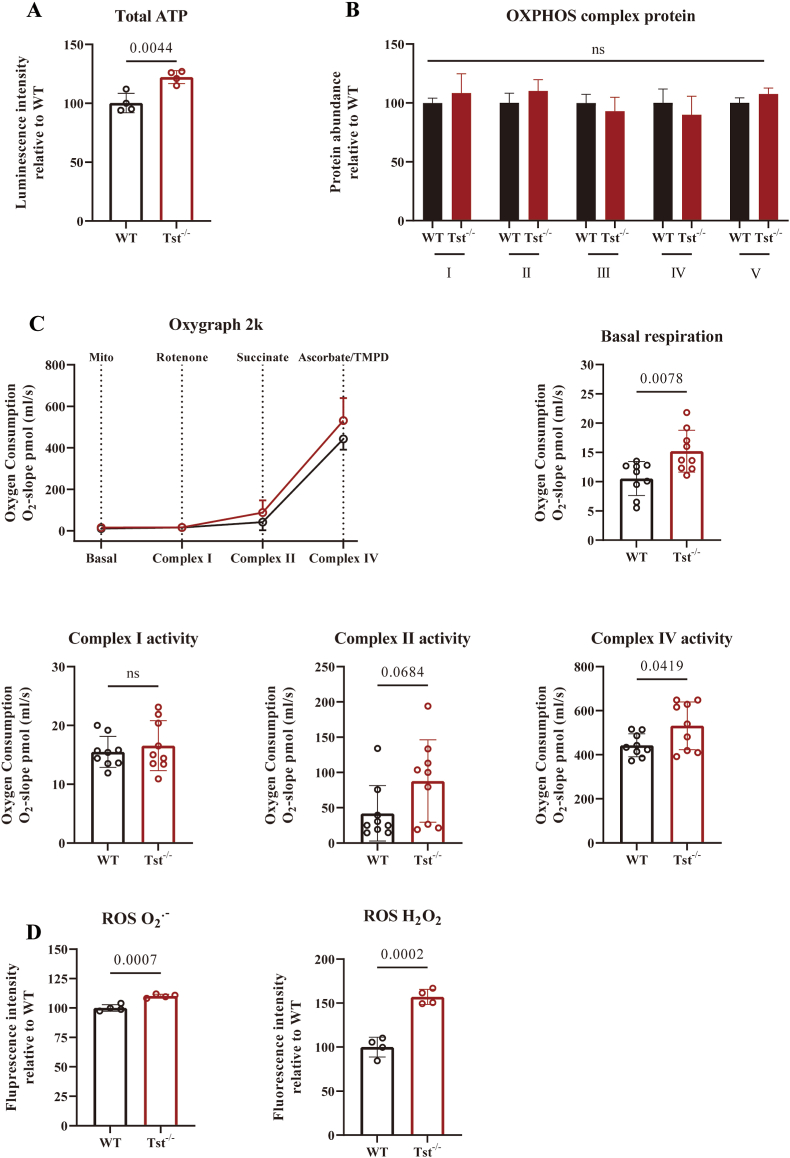
*Tst*^−/−^ mice brain cortexes display metabolism reprogramming. (A–D) Data expressed as mean ± SD, student *T* test. (**A)** Total ATP level of C57BL/6J (black) and *Tst*^−/−^ (red) mice cortical area (n = 4). (B) OXPHOS protein levels of two genotypes detected by OXPHOS cocktail antibody (n = 3). Quantification is normalized to ponceau stain. Immunoblot is displayed in Fig. S2. (C) Corrected oxygen consumption rate of 250 μg crude isolated mitochondria from mice cerebral cortices with various substrates. Respiratory capacity differences of basal respiration, Complex I, Complex II and Complex IV from C57BL/6J (black) and *Tst*^−/−^ (red) were analyzed. (n = 3 biological samples, independent experiments were repeated at least 3 times). (D) Relative ROS contents as O_2_^.-^ and H_2_O_2_ of C57BL/6J (black) and *Tst*^−/−^ (red) (n = 4). (For interpretation of the references to colour in this figure legend, the reader is referred to the Web version of this article.)

Since TST exists almost exclusively in mitochondria and is capable of modulating mitochondrial respiratory complexes, we examined the impact of *Tst* deletion on mitochondrial activity in the cortical brain areas. For that purpose, we performed total ATP measurements generated by OXPHOS via luminescence. Total ATP was increased by 22% in the *Tst*^*−/−*^ mice cortex compared with C57BL/6J mice (Fig. 2A). Next, we measured the protein levels of OXPHOS complexes, and found no significant differences between the two mouse genotypes (Fig. 2B; Fig. S2A). In contrast, basal respiration and complex IV activity represented by oxygen consumption rate were markedly increased in the cortex of *Tst*^−/−^ mice, while no significant differences in Complex I capacity were observed among either mouse genotypes (Fig. 2C). The increase of complex IV activity in the absence of TST indicates an impact of this enzyme on mitochondrial capacity. The function of TST as modifier of GSH to form glutathione persulfide (which is a more efficient ROS scavenger than GSH itself) has been well characterized [12,16]. We next determined the extent of O_2_-derived reactive species formation from OXPHOS. The O_2_^.−^ level was 10% higher in the cortex of *Tst*^−/−^ mice, while tissue levels of H_2_O_2_ were 57% higher in the cerebral region of *Tst*^−/−^ mice compared to C57BL/6J control mice (Fig. 2D).

#### 3.3.1 *Tst* deficiency reprograms the antioxidant defense against endogenous ROS

**Fig. 3:**
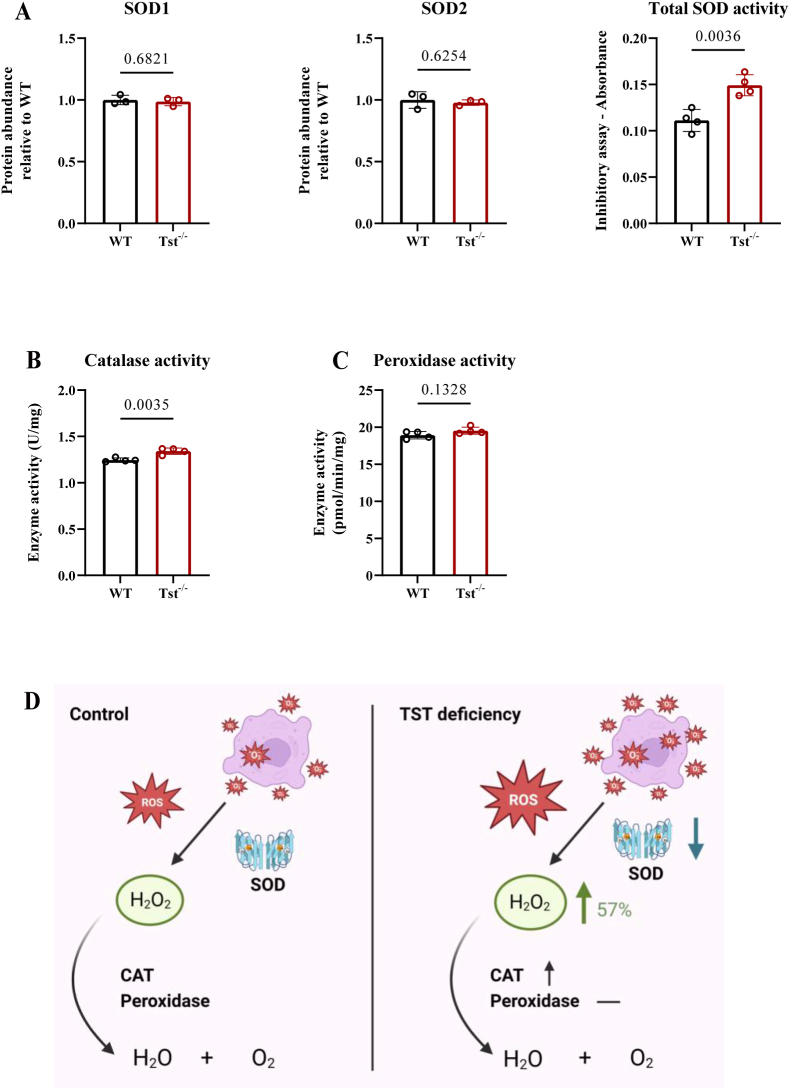
*Tst*^−/−^ mice cerebral cortical regions display reprogrammed antioxidant defense. (A–D) Data expressed as mean ± SD, student *T* test. (A) SOD protein expression (n = 3) and enzyme activity (n = 4) levels of C57BL/6J (black) and *Tst*^−/−^ (red) mice cortical area. Original immunoblots are presented in Fig. S3A. 2. (B) Catalase enzyme activity measurements in C57BL/6J (black) and *Tst*^−/−^ (red) mice (n = 4). (C) Peroxidase activity in C57BL/6J (black) and *Tst*^−/−^ (red) mice (n = 4). (D) Created with Biorender.com. Brief scheme of response to excessive ROS with or without TST presence. SOD1 and SOD2 are vital endogenous antioxidant elements for H_2_O_2_ catalyzation from O_2_^−^. (For interpretation of the references to colour in this figure legend, the reader is referred to the Web version of this article.)

To determine whether antioxidant expression/activity changed in the cortex of *Tst*^−/−^ mice, we checked both protein and total enzyme activity levels of superoxide dismutase 1 (SOD1) and superoxide dismutase 2 (SOD2). Both, SOD1 and SOD2 are vital endogenous antioxidant enzymes. The expression of SOD1 and SOD2 proteins was unchanged between the two groups, whereas total SOD enzyme activity showed a 34% decrease in the *Tst*^−/−^ mice (Fig. 3A; Fig. S3A). When H_2_O_2_ is excessively produced, catalase participates in H_2_O_2_ breakdown. The catalase activity in *Tst*^−/−^ was 7.6% higher relative to that observed in C57BL/6J mice (Fig. 3B). In addition, the total peroxidase from *Tst*^−/−^ mice exhibited unaltered activity compared with C57BL/6J mice (Fig. 3C). In the context of the RSI framework, we also measured nitric oxide synthase (NOS) activity and RNS (NO and ONOO^−^) production. NOS activity and NO content were similar in the brain cortex of either genotype (Fig. S3B and D), while ONOO^−^ amount in cortexes of *Tst*^−/−^ mice was 10% lower than in C57BL/6J mice (Fig. S3C). In summary, we found decreased SOD enzyme activity, increased catalase activity and unchanged peroxidase activity, when ROS is elevated due to the global loss of TST in murine brain tissue as schematically depicted in Fig. 3D.

**Fig. 4:**
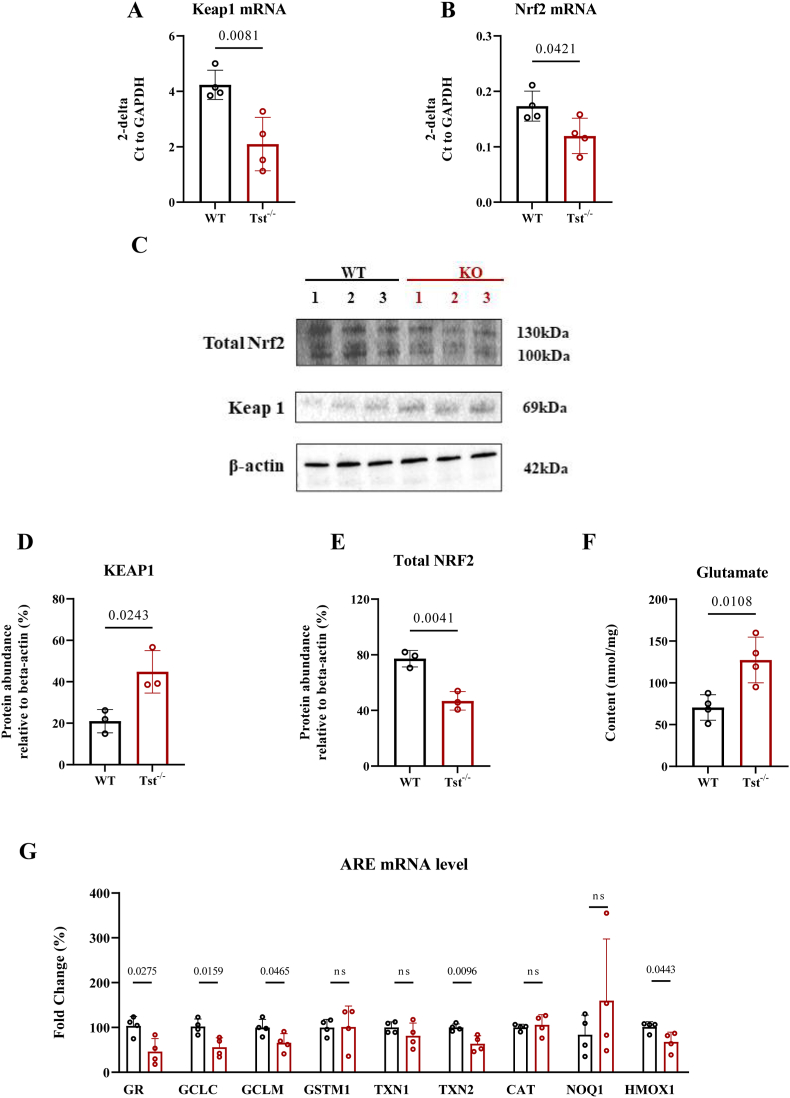
*Tst*^−/−^ mice brain cortexes display dysregulated Nrf2-keap1 signaling expression and increased glutamate content. A–B, D–G: Data expressed as mean ± SD, student *T* test. (A) Expression of *Keap1* mRNA in C57BL/6J (black) (n = 4) and *Tst*^−/−^ (red) (n = 4) mice levels of cortical area. (B) Expression of *Nrf2* mRNA in C57BL/6J (black) (n = 4) and *Tst*^−/−^ (red) (n = 4) mice levels of cortical area. (C) WB analysis of the two proteins. (D) Quantification of immunoblots of cerebral cortical area for NRF2 protein level in mice cortexes in the presence from C57BL/6J (black) (n = 3) and *Tst*^−/−^ mice (n = 3). (E) Quantification of immunoblots of cerebral cortical area for KEAP1 protein level in mice cortexes in the presence from C57BL/6J (black) (n = 3) and *Tst*^−/−^ mice (n = 3). (F) Glutamate content measurements in both genotypes (n = 4). (G) *Tst*^−/−^ mice brain cortexes display dysregulated ARE genes. P value of each gene is indicated between genotypes. All experiments were conducted with repeat number as 4 per genotype. (For interpretation of the references to colour in this figure legend, the reader is referred to the Web version of this article.)

### 3.3 *Tst* absence results in decrease of Nrf2-Keap1 pathway expression level and increased glutamate content

Activation of the Nrf2-Keap1 system results in an antioxidant defense response which is, at least in part, regulated by H_2_S and KEAP1 protein level [21,22,28]. To investigate whether *Tst* deficiency causes a dysregulation of Nrf2 signaling, qRT-PCR was conducted for genes regulated by antioxidant response elements (AREs) under control of NRF2. *Nrf2* and *Keap1* mRNA were significantly decreased in *Tst*^−/−^ cortical brain samples compared with C57BL/6J mice (Fig. 4A and B). NRF2 protein showed significant reduction and KEAP1 protein had an increased expression in *Tst*^−/−^ mice brain cortexes when compared to those of control mice (Fig. 4C–E). At the same time, different ARE genes including *Hmox1* and *Txn2* mRNA showed relative reduction in the case of *Tst* deficiency, while *Gstm1, Txn1, Noq1*and *Cat* revealed no significant difference between the two types of mice (Fig. 4G).

GCLC, GCLM and GR are important ARE genes for proteins protecting against oxidative stress. All these genes were found to be significantly reduced in the *Tst*^*−/−*^ brain (Fig. 4G). Since both GCLC and GCLM are critical for *de novo* GSH biosynthesis we also measured glutamate content and found a 1.8-fold increase in *Tst*^−/−^ compared to wildtype mice, corroborating the effect of *Tst* deletion on this important antioxidant pathway (Fig. 4F).

### 3.4 *Tst*^*−/−*^ mouse brain displayed deteriorated antioxidant capacity when facing paraquat-induced oxidative stress

**Fig. 5:**
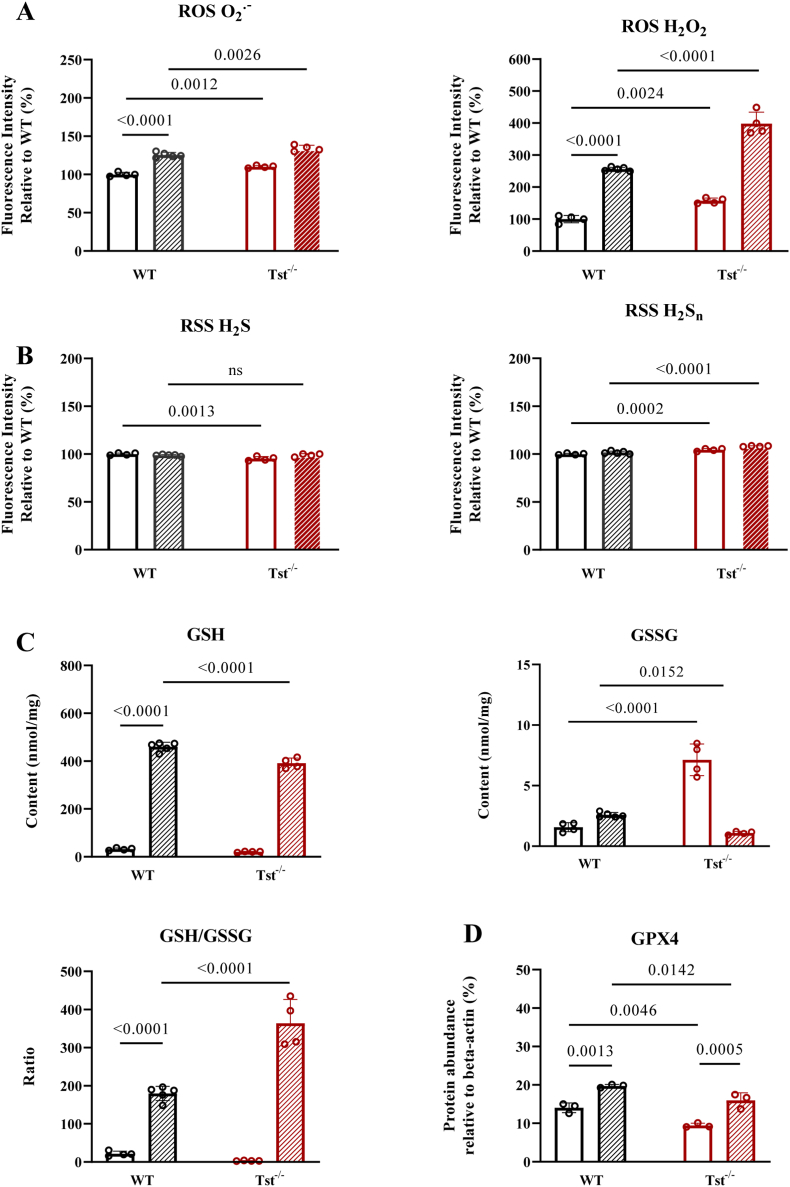
PQ challenge exacerbated Tst^−/−^ mice brain cortexes disrupted antioxidant capacity. (A–D) Data are expressed as mean ± SD, one-way ANOVA test. (A) Relative ROS contents as O_2_^−^ and H_2_O_2_ of mice brain cortexes in the presence (hatched pattern) or absence (no pattern) of 25 mg/kg 24 h from C57BL/6J (black) (n = 4–5) and *Tst*^*−/−*^ mice (n = 4). (B) Relative H_2_S and H_2_S_n_ concentrations of mice brain cortexes in the presence (hatched pattern) or absence (no pattern) of 25 mg/kg 24 h from C57BL/6J (black) (n = 4–5) and *Tst*^*−/−*^ mice (n = 4). (C) GSH and GSSG contents in mice cortexes in the presence (hatched pattern) or absence (no pattern) of 25 mg/kg 24 h from C57BL/6J (black) (n = 4–5) and *Tst*^*−/−*^ mice (n = 4). (D) Quantification of immunoblots of cerebral cortical area for GPX4 protein level in mice cortexes in the presence (hatched pattern) or absence (no pattern) of 25 mg/kg 24 h from C57BL/6J (black) (n = 3) and *Tst*^*−/−*^ mice (n = 3). The immunoblots are shown in Fig. S4.

In order to show the effects of TST loss when facing oxidative stress, we analyzed several oxidative stress markers including ROS, RSS, GSH/GSSG contents and GPX4 protein abundance. Under basal TST deficiency condition, ROS showed a significant increase in the *Tst*^*−/−*^ mouse brains. Upon challenging both genotypes of mice with 25 mg/kg paraquat (PQ) for 24 h, both genotypes of mouse brain cerebral cortices showed significantly increased ROS compared to their basal levels. Cerebral cortex of *Tst*^*−/−*^ mice showed 1.5-fold increase of H_2_O_2_ compared to those of C57BL/6J mice (Fig. 5A). As for reactive sulfur species, brain cortexes of *Tst*^*−/−*^ mice showed similar amounts of H_2_S in the cortical brain area compared to C57BL/6J mice. Upon PQ challenging, there was no difference in both H_2_S and H_2_S_n_ intensity between the two genotypes (Fig. 5B).

The parameters relevant to the GSH content were also assessed. Following paraquat stimulation, both mouse genotypes had significantly higher GSH content compared to their basic levels in brain tissue. However, GSH amount generated in the brain cortex of *Tst*^*−/−*^ mice was less than that of C57BL/6J mice. In contrast to the accumulation of GSSG in basal cerebral cortical area of *Tst*^*−/−*^ mice, PQ-treated *Tst*^*−/−*^ mice also had relatively lower GSSG compared to C57BL/6J mice. Therefore, in the cerebral cortex of PQ-treated mice the ratio of GSH/GSSG showed a significant increase when compared with C57BL/6J mice (Fig. 5C). Furthermore, we tested GPX4 protein expression and found that GPX4 had higher levels in PQ injected animals in response to acute redox damage in both mouse genotypes. In the mouse brain of *Tst*^*−/−*^ mice, GPX4 protein level was slightly lower than in C57BL/6J mice (Fig. 5D). In summary, this additional challenge experiment showed an overall deteriorated antioxidant capacity in murine cerebral cortices in conditions of global TST deficiency.

## 4 Discussion

TST plays an important role in the oxidation of the endogenous gasotransmitter hydrogen sulfide, complementing the action of sulfide quinone oxidoreductase (SQOR) and persulfide dioxygenase (ETHE1/PDO) [9,42]. Whether or not TST also contributes to antioxidant protection in the brain has not been investigated before. In *Tst*^−/−^ mice, circulating thiosulfate and sulfide level were markedly elevated, while cerebral cortex displayed similar steady-state levels of sulfide and thiosulfate, as observed in the liver [43]. This indicates that the response to *Tst* knock-down is organ specific in this chronic but viable sulfide elevation model. Starting from organ specificity, the free thiol antioxidant system of brain and liver revealed opposite responses in terms of GSH content. In *Tst*^−/−^ mice, GSH was decreased in the cerebral cortex, while there was no difference in GSH content between the two genotypes in the liver [43]. Since NADPH/H^+^ dependent glutathione reductase (GR) is important for the recycling of GSSG to GSH [44], its down-regulation in the brain of *Tst*^−/−^ mice explains the alterations in GSSG levels observed. In support of an activation role of TST on ROS scavengers such as GSH [15], *Tst*^−/−^ mice showed a lower GSH/GSSG ratio and higher ROS levels, indicating a redox signaling imbalance along with lower GPX4 and GR expression. Meanwhile, the *Mpst* transcription level of brain and liver in *Tst*^−/−^ mice were reduced. In contrast, the liver functions as a major detoxification organ, and despite lower mRNA for *Mpst* protein levels were upregulated, perhaps for compensatorily enhanced sulfide removal [43], while MPST protein significantly decreased in the brain cortex and in mitochondria in *Tst*^−/−^ mice.

TST interacts with iron-sulfur cluster proteins including Complex I (NADH dehydrogenase), Complex II (succinate dehydrogenase) and Complex III (Cytochrome *bc*1) of the mitochondrial respiratory chain) in oxidative pathway [45]. TST directly interacts with Complex I via sulfide transfer.^6^In addition, TST transfers sulfane sulfur to Complex II and modifies its iron-sulfur structure via alteration of its phosphorylation status. Those functions led us to explore the metabolic phenotypes of the brain tissues of C57BL/6J and *Tst*^−/−^ mice. The unexpected finding of mitochondrial activity remodeling was supported by several observations, including unchanged OXPHOS protein expression, increased basal respiration capacity and Complex IV activity in the presence of ascorbate and TMPD. However, the cerebral cortex of *Tst*^−/−^ mice produced more ATP, and higher amounts of ROS compared to C57BL/6J control mice. ATP, the universal energy currency, is mainly synthesized in mitochondria by OXPHOS. The representative role of Complex IV as regulatory center of OXPHOS limits the rate of respiratory chain reaction [46]. Hence, our finding of enhanced Complex IV activity is in line with the observation of enhanced ATP levels in *Tst*^−/−^ mice.

ROS are generated during the incomplete reduction of O_2_ [47,48]. In mitochondria, the major sources of O_2_^•^−are the OXPHOS Complexes I and III [20,49]. O_2_^•^−will be further processed quickly to H_2_O_2_ via SOD1 and SOD2 [50]. In the absence of TST, the significant inhibition of total SOD activity can be explained by excessive H_2_O_2_ in the brain cortex, which could possibly slow down H_2_O_2_ production as oxidative distress response. The metabolism of H_2_O_2_ to H_2_O is catalyzed by catalase, glutathione peroxidase, and peroxiredoxin 1 [51]. Compared to O_2_^•^^−^, the steadier feature of H_2_O_2_ allows for mitochondrial modulation of the cellular antioxidant system [52]. The observed alterations in ROS metabolizing enzymes indicate the importance of TST in overall antioxidant capacity to protect the brain.

Our study of the Nrf2 pathway in *Tst*^−/−^ mice highlights a potent interaction between Nrf2 signaling and the GSH antioxidant system [[53], [54], [55], [56]]. Importantly, Nrf2 is a major transcriptional factor regulating multiple processes related to GSH synthesis and regeneration [57,58]. Beyond GSH content change, the transcriptional and protein levels of Nrf2 appear to be lower and its intracellular inhibitor Keap1 had higher protein expression in the brains of Tst^−/−^ mice. A possible reason for the fact that TST deletion causes disrupted Nrf2-Keap1 signaling, might be found in the abnormal cysteine metabolism within the redox sensor protein KEAP1. TST's main function is sulfide oxidation as cysteine metabolism downstream, and KEAP1 protein contains 25 cysteines within mouse homologue, thus accumulated KEAP1 were detected in the brain of *Tst*^*−/−*^ mice, consequently lower amount of Nrf2 protein was released from KEAP1 ubiquitin E3 ligase adapter, which further influenced the ARE gene transcription process [22,59,60]. In light with reduced transcription of *Keap1*, we found an increased KEAP1 protein level, and we suggest a post-transcriptional cellular redox-sensing mechanism as operative.

Similar reduction in Nrf2 activation in *Tst*^−/−^ mice using transcription factor binding site (TFBS) enrichment analysis were found earlier [43]. Additionally, glutamate-cysteine ligase catalytic (GCLC) and modifier (GCLM) subunits together catalyze the rate-limiting step in GSH biosynthesis from glutamate under conditions of oxidative distress [26]. *Nrf2*^*−/−*^ mice exhibited a decrease in GSH content and GCL levels [61], which supported our results on the brains of *Tst*^−/−^ mice. The decrease in Nrf2 mRNA and protein translate into a reduced function of the GSH antioxidant system. Moreover, the decrease in Nrf2-mediated free thiol antioxidants influences various cellular processes, ranging from synthesis, oxidation and reduction of GSH. Among the heterogeneity of brain cell types, the expression of Tst mRNA is known to be highest in astrocytes (https://www.proteinatlas.org/ENSG00000128311-TST/single+cell+type/brain). Astrocytes comprise the majority of glial cells in the mammalian central nervous system (CNS) and serve as major provider of GSH for neurons [62,63]. Astrocytes contribute to neuronal activity, and their interaction play distinct, cooperative roles in neuronal redox homeostasis. Astrocytes could initiate and promote an increase in gene expression of Nrf2-mediated antioxidant pathway in response to mild oxidative stress, which results in the synthesis of GSH using glutamate [64]. When astrocytes function abnormally, glutamate, which is an excitatory neurotransmitter, will accumulate to an excitotoxic level and contribute to harmful effects on neurons, eventually causing impaired brain function [65]. Thus, any deficiency in astrocyte capacity to produce GSH (or reduce GSSG) will render neurons particularly vulnerable to conditions or insults known to be associated with enhanced oxidative stress [[66], [67], [68], [69], [70]]. Both oxidative stress and elevated glutamate contribute and are associated with brain pathologies [65]. The decreased Nrf2 expression, reduced GSH and increased glutamate content, together with an altered ROS landscape in the CNS, secondary to a loss of TST are therefore key observations of the present study.

Relevant to Nrf2 signaling, another important thiol-dependent antioxidant system is the thioredoxin (TXN) system [71]. TXN2 is a mitochondria-specific protein, whereas TXN1 is found in the cytosol, both of them operate using NADPH as cofactor [72]. Down-regulated *Txn2* mRNA level observed in the cerebral cortex of *Tst*^−/−^ mice, could be explained by loss of TST's function to interact with mitochondrial NADH dehydrogenase (Complex I) and other iron-cluster proteins [6]. Our findings provide a better understanding of the role of TST in linking Nrf2-keap1 signaling and thiol antioxidant pathways.

When challenging mice with paraquat-induced oxidative stress, the ROS production in *Tst*^*−/−*^ mouse brain was dramatically increased due to the hampered antioxidant capacity, indicating the importance of the interaction of TST with the antioxidant pathway. The nature of GSH is to provide a protective effect on free radical scavenging and H_2_O_2_ detoxification [73] therefore, after stimulation, increased GSH content was detected in both genotypes, although *Tst*^*−/−*^ mouse brain had lower GSH levels than control mice. Unlike the basal condition, PQ-treated mice biosynthesized less GSSG when the brain tissue of *Tst*^*−/−*^ mice was compared to the WT animals. PQ injection also induced an increase in GPX4 protein expression in response to excessive oxidative distress. GPX4 protein in *Tst*^*−/−*^ mice was slightly lower than in C67BL/6J mice. In summary, this additional challenge experiment showed an overall deteriorated antioxidant capacity in murine cerebral cortex under the model of global TST deficiency.

In the context of brain function, both mitochondria and NRF2 functions are particularly important because the brain is highly susceptible to oxidative distress due to its high metabolic rate and relatively low levels of antioxidants compared to other tissues [74]. During oxidative stress, structural and functional damage occurs to neurons and disrupts the normal actions of neurotransmitters, leading to altered signaling from neurons. This can lead to impaired cognitive function, affect behavior, and contribute to the development and progression of neurological and neurodegeneration diseases [75]. Taken together, our data unmask several tissue-specific mechanisms linked to TST function that are related to mitochondrial and Nrf2-Keap1 signaled antioxidant functions, and the role of TST in maintaining the health and function of brain tissues.

## 5 Conclusion

Our study on mouse cerebral cortex revealed that TST deficiency promotes a dysregulation of the reactive species interactome through both generation of ROS and RSS, coupled with mitochondrial OXPHOS remodeling. In addition, TST deficiency elicits the dysregulation of thiol-dependent antioxidant systems with altered GSH levels and aberrant Nrf2 pathway expression. Moreover, the absence of TST is characterized by a deteriorated antioxidant buffering ability in the murine brain cortex when challenged with a redox cycler as demonstrated by paraquat *in vivo*.

## Funding

Y.L. was supported by 10.13039/501100004543China Scholarship Council (Grant No.: 202008520033). Z. M. Al-Dahmani was supported by the 10.13039/501100003971Islamic Development Bank. A.M.D. was supported by a Rosalind Franklin Fellowship co-funded by the 10.13039/501100000780European Union and the 10.13039/501100001721University of Groningen. S.M. was supported by a 10.13039/501100000274British Heart Foundation 4Y PhD Scholarship (FS/4yPhD/F/20/34,126). L.C was supported by the 10.13039/501100004794CNRS.

## Contribution statement

YL, AM and HvG designed and conceptualized the study, wrote and revised the manuscript. YL conducted parts of research, LC designed and performed parts of study, edited the manuscript. YL and LC analyzed the data. ZMM conducted parts of experiments. SM, NZMH and NMM performed experiments on the animal model, provided samples, and edited the manuscript. MG and MF revised the manuscript. AM and HvG supervised this study.

## Declaration of competing interest

The authors declare that they have no known competing financial interests or personal relationships that could have appeared to influence the work reported in this paper.

## Data Availability

Data will be made available on request.
