# Supplementary material for: Thiosulfate sulfurtransferase deficiency promotes oxidative distress and aberrant NRF2 function in the brain
Source: Redox Biol. 2023 Nov 19;68:102965. doi: 10.1016/j.redox.2023.102965 (PMC10701433; doi:10.1016/j.redox.2023.102965)
Supplement: Multimedia component 1 [file mmc1.docx]

**Fig S1.** **Supplementary Figures to Figure 1.**


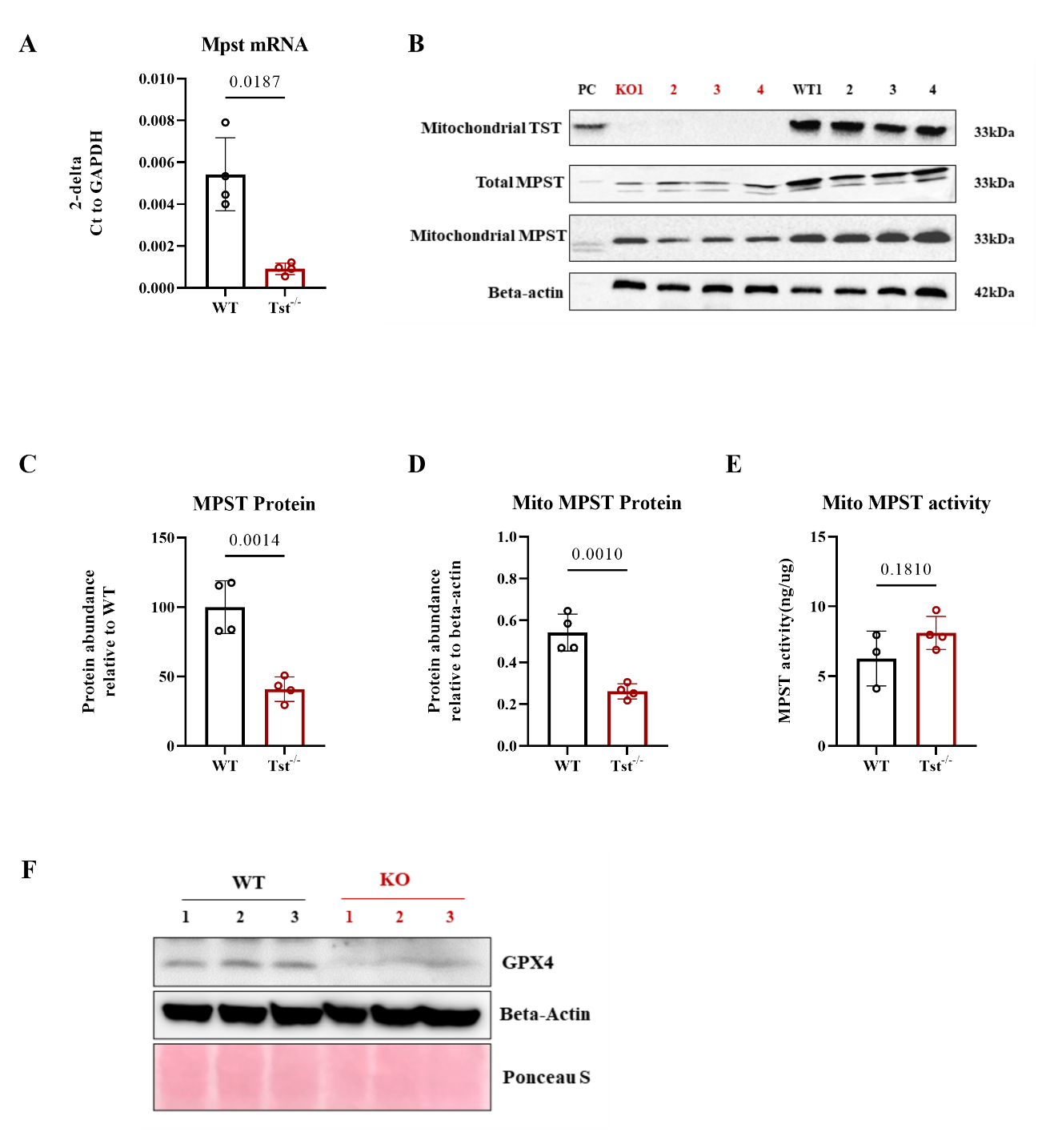


**Fig. S1. Supplementary figures related to Figure 1.** (A) Immunoblots of GPX4 protein related to **Fig.1D**. (B) Mpst mRNA quantified by real time PCR from cortices of C57Bl/6J (black, n = 4) and *Tst*^-/-^ (red, n = 4) mice. (C) Representative immunoblots for MPST from isolated mitochondrial and total brain cortical tissue. (D) Quantification of western blots for MPST from isolated brain cortex of C57Bl/6J (black, n = 4) and *Tst*^-/-^ (red, n = 4) mice. (E) Quantification of western blots for MPST from isolated cortical mitochondria of C57Bl/6J (black, n = 4) and *Tst*^-/-^ (red, n = 4) mice. (F) Quantification of MPST activity in mitochondrial brain cortexes. Data are represented as mean ±SD. Significance was calculated using un-paired two-tailed student’s t-test.

**Figure S2. Supplementary figures to Figure 2.**

**
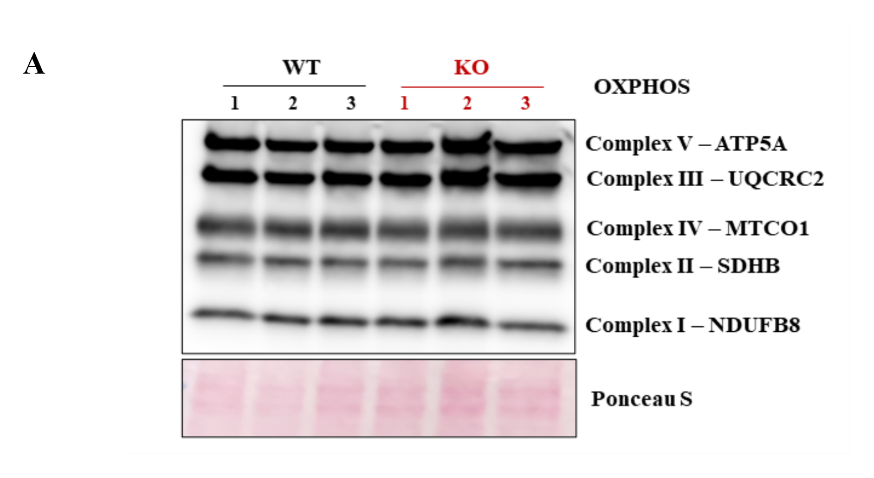
**

**Fig. S2. Supplementary figures related to Figure 2.** (A) Immunoblots of OXPHOS complexes from isolated brain cortexes of C57Bl/6J (black, n = 3) and *Tst*^-/-^ (red, n = 3) mice.

**Figure S3. Supplementary figures to Figure 3.**


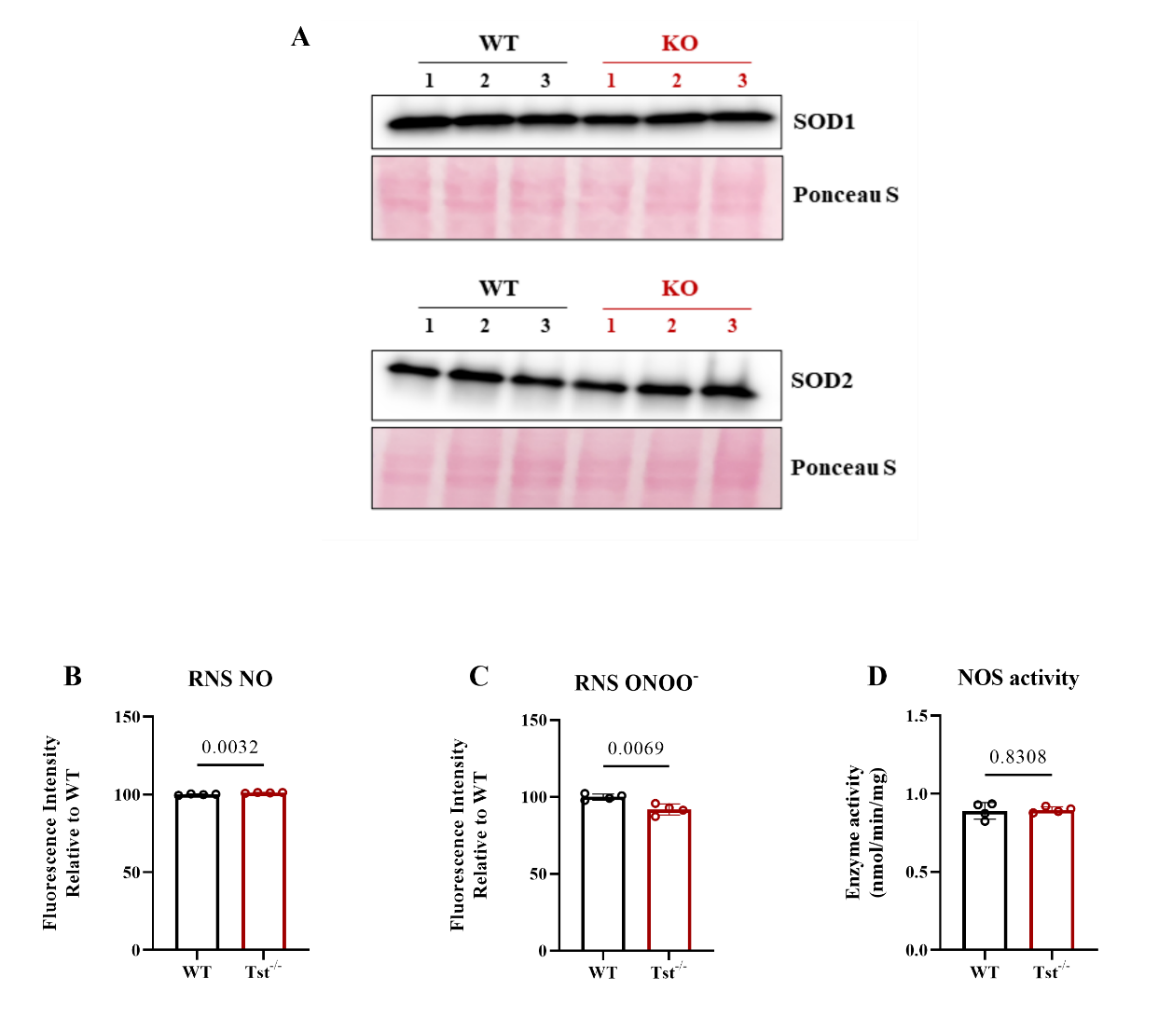


**Fig. S3. Supplementary figures related to Figure 3.** (A) Immunoblots of SOD1 and SOD2 proteins from isolated brain cortexes of C57Bl/6J (black, n = 3) and *Tst*^-/-^ (red, n = 3) mice. (B) Relative RNS content as NO of C57BL/6J (black) and *Tst*^-/-^ (red) mice (n=4). (C) Relative RNS content as ONOO^-^ of C57BL/6J (black) and *Tst*^-/-^ (red) mice (n=4). (D) Nitric oxide synthase (NOS) activity measurements in C57BL/6J (black) and *Tst*^-/-^ (red) mice (n=4).


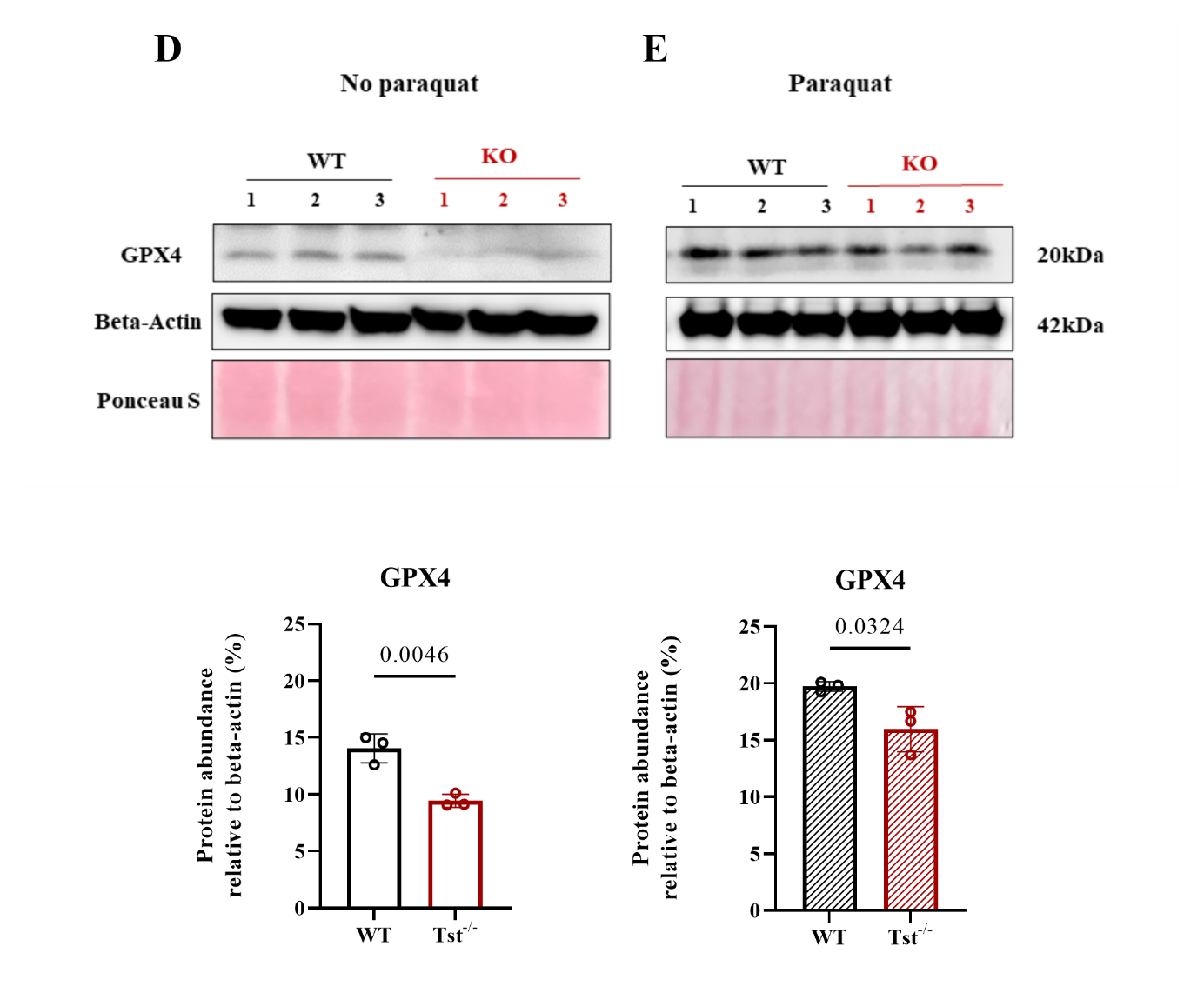


**Fig. S4. Supplementary figures related to Figure 5.**

**Supplementary Table 1. Sequences of primers used in real-time PCR.**

Forward and reverse primers used in real-time PCR for the Nrf2-keap 1 pathway detection (mouse). All the primers are ordered from Integrated DNA Technologies (Belgium).

| Name | Primers-F (5’-3’) | Primers-R (5’-3’) |
| --- | --- | --- |
| TST | GGAGCCCGGATATAGTAGGACTAGA | TTCGTCAGGAAGTCCATGAA |
| MPST | TCACAGCCGCTGAAGTTACTG | CAGCATGTGGTCGTAGGGG |
| GCLC | GGGGTGACGAGGTGGAGTA | GTTGGGGTTTGTCCTCTCCC |
| GCLM | AGGAGCTTCGGGACTGTATCC | GGGACATGGTGCATTCCAAAA |
| GR | GACACCTCTTCCTTCGACTACC | CCCAGCTTGTGACTCTCCAC |
| GSTM1 | ATACTGGGATACTGGAACGTCC | AGTCAGGGTTGTAACAGAGCAT |
| NFE2L2 | TCTTGGAGTAAGTCGAGAAGTGT | GTTGAAACTGAGCGAAAAAGGC |
| CAT | AGCGACCAGATGAAGCAGTG | TCCGCTCTCTGTCAAAGTGTG |
| NQO1 | ATGGGAGGTGGTCGAATCTGA | GCCTTCCTTATACGCCAGAGATG |
| HMOX1 | AAGCCGAGAATGCTGAGTTCA | GCCGTGTAGATATGGTACAAGGA |
| KEAP1 | TGCCCCTGTGGTCAAAGTG | GGTTCGGTTACCGTCCTGC |
| TXN1 | CATGCCGACCTTCCAGTTTTA | TTTCCTTGTTAGCACCGGAGA |
| TXN 2 | TGGGCTTCCCTCACCTCTAAG | CCTGGACGTTAAAGGTCGTCA |
| GAPDH | TTT GAT GTT AGT GGGG TCT CG | AGC TTG TCA ACGGG AAG |
